# Health coaching provided by registered nurses described: a systematic review and narrative synthesis

**DOI:** 10.1186/s12912-021-00594-3

**Published:** 2021-05-10

**Authors:** Jennieffer A. Barr, Lily P. Tsai

**Affiliations:** College of Nursing and Midwifery, Charles Darwin University, Darwin, Casuarina, Northern Territory Australia

**Keywords:** Registered Nurses; health coaching, Systematic review, Narrative synthesis, Chronic illness

## Abstract

**Aims:**

The aim of this systematic review and narrative synthesis was to identify how and why health coaching is delivered by Registered Nurses.

**Design:**

Systematic review and narrative synthesis.

**Data sources:**

Articles were identified through a search of CINAHL, Medline, Scopus, and PsychINFO databases. Articles published in English between 2010 and 2021 were included.

**Review Methods:**

Quality appraisal of relevant literature was independently undertaken by two authors to assess for risk of bias. The Critical Appraisal Skills Program (CASP) was used to appraise quality of potential papers.

**Results:**

A main purpose of coaching by Registered Nurses is to optimise patient self-care. How coaching was conducted varied across studies, with the most common coaching approaches via telephone or online. Majority of studies highlight some effectiveness of coaching by nurses; however, some results were inconclusive. Health coaching generally reduced mental distress. Other benefits reported by patients included reduced pain and fatigue. Outcomes for changing lifestyle behaviours were mixed. However, for health coaching to be efficient greater evidence is needed to determine length of time to use coaching, number of habits to focus on to produce change, and to determine best training for coaches.

**Conclusions:**

Registered Nurses are most suitable for implementing health coaching for self-care, including preventing and managing chronic illness and recovering from situations like post-surgical needs. Nurses already promote health, and therefore, are skilled in educating people in self-care. Coaching is an additional strategy for motivating, targeting and assessing progress of self-care. Extending the scope of nursing practice to routinely coach in self-care would be ideal.

## Introduction

Health coaching is the latest tool being implemented in contemporary health care for managing chronic illness. Previously coaching has been used in sport and more recently in business for motivating people to achieve their personal and professional goals [1]. Coaching is now being used in health care by a range of health professionals.

Registered Nurses are well placed to provide health coaching. Coaching can be used to prevent ill health as well as reduce the impact of symptoms when living with a chronic condition. However, currently it is not known how coaching is being applied in nursing practice.

## Background

The general literature in coaching is broad but does provide some important principles for the background of this paper. First there is an array of different ways to coach, therefore defining what is meant by coaching is important in any discussion about this area. Coaching differs to mentoring where a mentor is the ‘expert’ in the area [2]. Alternatively, a coach does not have to be an ‘expert’ in the area and may not give specific solutions. As a mentor, instruction and goal setting is typical [2]. Sports coaching also typically includes goal setting, skill development and competency [3, 4].

Within the literature, health coaching has been defined and described in a variety of ways. Health coaching is a person-centred, collaborative relationship between coach and coachee that involves the process of health promotion and education [1, 5, 6]. Health coaching aims to motivate the client to achieve personally identified health-related goals set during the coaching sessions [1, 7–9]. Health coaching assists the client to navigate through options, make choices, plan and identify challenges, and facilitate the changing process relating to their health behaviours [7, 10] leading to disease management [11].

In the quest to find one definition of health coaching relevant specifically for nurses, the work written by [6] during collaboration of two highly regarding professional bodies of nursing, the International Council of Nurses and Sigma Theta Tai International was found to be relevant. This definition [6] was chosen because:

- the definition is specific to nursing, and
- it provides features that were useful to guide this inquiry.

Palmer et al. [6] argues that coaching includes:

*…a collaborative relationship undertaken between a coach and a willing individual, the client. It is time-limited and focused and uses conversations to help clients achieve their goals. It demands skill on the part of the coach in facilitating meaningful conversations and letting the client “lead.” Leading starts when the coaching conversation begins and new actions and new practices are always the final stage of a successful coaching conversation* [12].

According to the above definition [6], the values of the profession of nursing typically complement the act of coaching. Building rapport [13, 14], actively listening [15, 16], respecting and working with patients [17, 18] and responding to individual needs [19] are all values denoted in nursing and coaching. The principle proposed by [12] above that a client should “lead” is an example of person-centred care. Like coaching conversations, health assessment conversations should allow the person to explore and state what needs should be met. The nurse, like the coach will go beyond this initial conversation and then explore what else the client needs.

A number of theories used by nurses are complementary to coaching interventions. The obvious theory that could be shared in both nursing and coaching is the philosophy of holistic care. Holistic care is defined as “*behaviour that recognizes a person as a whole and acknowledges the interdependence among one’s biological, social, psychological, and spiritual aspects*” [20]. Therefore, holistic care aims to meet all human needs according to the importance to the patient [21]. Similarly, coaching will also aim to meet human needs that are important to the client.

Another theory, Orem’s Model of Nursing [22], focuses on the principle of patients being as independent as possible with their own self-care needs. Orem’s Model of Nursing can also be used in conjunction with coaching [23]. Coaching provides a platform for nurses to build on the strength of individuals, which is a similar sentiment found in the theories like holistic care and Orem’s Model of Nursing. Considering the current practice of Registered Nurses, how coaching is different to typical practice was an important consideration during this inquiry. The answer of how coaching differs to typical nursing is the focus on patient transformation [24] aid the understanding of achieving patient transformation when they defined nurse coach as “*a Registered Nurse who integrates coaching competencies into practice to facilitate a process of change or development with individuals or groups to enhance their growth*.” Effective change must evolve from within individual person; therefore, the nurse coach works with the person, knowing that change will require an integration of body, mind, emotion, spirit, and environment [24]. As noted above, thinking about the person has a holistic being influenced by environment is not a new principle to nurses and has been applied in nursing practice for many decades. However, [24] do place an emphasis on facilitating change which is paramount in coaching but may not always be the focus when delivering nursing care.

The principle of “change” was noted in Maslow’s theory which aims to support a person to reach the maximum way of being; self-actualization [25]. Maslow’s theory has extensively been used to guide nursing practice as it is in transformative coaching. Transformative coaching encourages people to reach their potential [26].

Whilst nurse coaching does align well with the traditional principles of nursing what is not yet known is how health coaching is provided by Registered Nurses. This stimulated this inquiry and the question was asked: How do nurses coach and why is coaching used?

## The review

### Aim

The aim of this systematic review and narrative synthesis was to identify, access, and summarize evidence related to how and why health coaching is implemented by Registered Nurses.

### Design

This systematic review was designed and reported based on the international guideline: The Preferred Reporting Items for Systematic Reviews and Meta-Analyses (PRISMA) [27]. The narrative synthesis is a strategy that examines the words from all studies to explain findings. A thematic analysis as outlined by Braun and Clarke [28] was used to summarize findings from qualitative, quantitative and mixed methods studies.

### Search methods

Articles were identified through a search of CINAHL, Medline, Scopus, and PsychINFO databases. The search terms employed were: ‘nurse’, ‘health’, ‘coaching’ and Medical Subject Heading (MeSH) terms related to ‘coaching’. For a paper to be considered, the focus of coaching provided by Registered Nurses to patients or clients was paramount. Empirical studies written in English were included in the search. The results from each database were saved in a specifically designated folder of that database, followed by hand searching for duplicates to be removed. Titles and/or abstracts of studies were retrieved using this search strategy. Hand searching of reference lists were also used to screen and identify studies that may have been missed. All papers were screened by two authors to identify studies that potentially met the inclusion criteria.

### Search outcomes

**Fig. 1:**
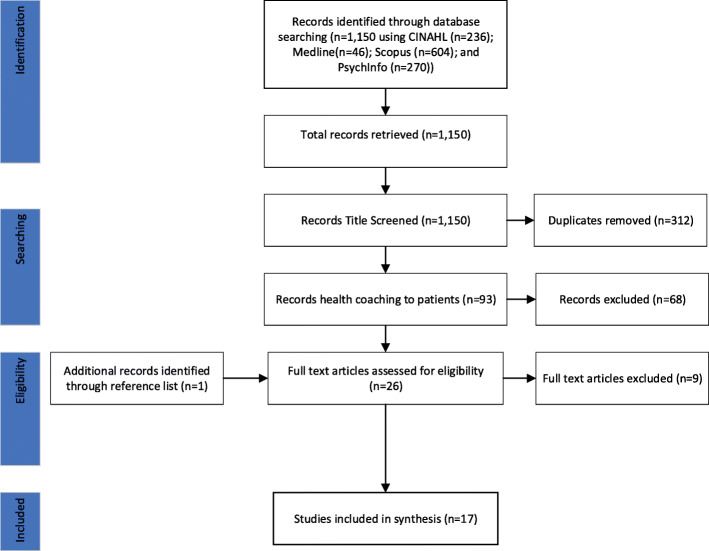
PRISMA diagram

The PRISMA process for reporting and the results of the searches was used ([27]; Fig. 1). The database searches revealed total of 1150 hits. First, these articles were screened for the duplicates which removed 312 duplications. Then, remaining 93 studies were screened for its relevance which removed 68 articles. A total of 27 full-text studies were assessed against following inclusion criteria:

1. conducted health coaching;
2. health coaching intervention was provided by Registered Nurses;
3. health coaching provided to patients or clients; and
4. paper was published in English from 2010 to February 2021. This time frame was selected as the word ‘health coaching’ started to appear in empirical studies around this period.

Systematic, integrative, and other literature reviews were also included if they met the inclusion criteria. A further ten studies were excluded. The main reason for exclusion was that the study did not specify the health coaching was delivered by Registered Nurses. Another study was identified through searching reference lists of included studies. This resulted in a total of 17 studies that were included for full review and synthesis.

### Quality appraisal

Quality appraisal of the relevant literature were independently undertaken by two authors to assess for risk of bias. The Critical Appraisal Skills Programs (CASP) were used to appraise quality of potential papers. CASP was implemented to assess methodological validity of each paper prior to inclusion in the review [29]. A total of 10 questions systematically appraised each study. The score meanings included: scored 0 (contained no information), 1 (minimal information), or 2 (fully addressed) [30]. A possible total score for a study is 20 points. Both authors independently rated each study and then compared results. Any disagreements that arose between the reviewers were resolved through discussion. All authors had noted they had gained ethical approval. To avoid publication bias, each of these studies were assessed for overlap between sub-studies; however, none of the data appeared to repeat.

High quality studies are those with a CASP score of 17 or more. In this inquiry three studies were scored 17 or more. Majority of studies (*n* = 11) were written in moderate quality, with a score between 14 and 16. Three studies were given low quality scores of 13 or less. The main reasons for these low scores included limited details on recruitment strategies, ethical consideration and data collection. Following a review against inclusion criteria and CASP rating, all 17 studies were deemed suitable to be included in the final systematic review (Table 1).

**Table 1:** Articles included in the review and CASP scores

References & location of study undertaken	Aim	Health areas	Design	Sample	Main outcome variables/ scales used	Primary result	CASP score
[31] Australia	To evaluate the effectiveness of goal focused telephone coaching by practice nurses in improving glycaemic control in patients with type 2 diabetes in Australia.	Chronic illness	Prospective, cluster RCT with GP as the unit of randomisation	59 GP clinics, 437 patients	Mean absolute change in HbA1c level	At 18 months follow-up, the effect on glycaemic control did not differ significantly. The median number of coaching sessions received by the 236 intervention was 3, of which 25 % did not receive any coaching sessions.	16
[32] (related to [33]) UK	To explore experiences about how health coaching motivated behaviour change.	Chronic illness	Qualitative	10 control participants and 20 intervention participants	-	Participants positively enacted behaviour change to become more physically active. Participants took advantage of environmental affordances to pull themselves toward activity targets or relied on being pushed to be more active by the health coach or significant others. Behaviour change was maintained where efforts to be more active were built into the everyday lifeworld of participants.	16
[34] USA	To explore different types of successes experienced by adults with type-2 diabetes participating in a health technology and nurse coaching clinical trial.	Chronic illness	Qualitative	132 cases reviewed	Participants survey results Notes by nurse coaches	(1) change in health behaviours; (2) change in mindset or awareness; (3) change in engagement with healthcare resources; (4) change in physical or emotional health; and (5) change in health indicators.	13
[35] USA	To evaluate a behaviour support intervention for patients with poorly controlled diabetes.	Chronic illness	RCT with repeated measures	201 patients with poorly controlled type2 diabetes mellitus	HbA1c value Participant’s review of intervention material Diabetes Knowledge Test Summary of Diabetes Self-Care Activities Measure	There was a significant overall reduction in mean haemoglobin A1c value from baseline to 6 months but differences between groups, diabetes knowledge, and selfcare were not significant.	16
[33] UK	To evaluate the effectiveness of telephone health coaching delivered by a nurse to support self-management in a primary care population with mild symptoms of chronic obstructive pulmonary disease (COPD).	Chronic illness	RCT	71 GP clinics, 577 patients with dyspnoea	Quality of life (St George’s Respiratory Questionnaire)	No difference in SGRQ-C total score at 12 months. Compared with patients in the usual care group, at six months follow-up, the intervention group reported greater physical activity, more had received a care plan, rescue packs of antibiotics, and inhaler use technique check.	18
[36] USA	To test the hypothesis that ambulatory arthroscopic surgery patients who receive a nurse-coached telephone intervention will have significantly less symptom distress and better functional health status than a comparable group who receive usual practice.	Post-surgery	RCT	102 participants (52 intervention; 50 usual care)	Symptom distress scale Medical Outcomes Study 36-item short-Form health survey general health perceptions Mental health subscales	Intervention participants had significantly less symptom distress at 72 h and 1-week post-surgery and significantly better overall physical and mental health at 1-week post-surgery.	14
[37] USA	To determine if metabolic risk factors can be stabilized or improved with weekly motivational interviewing/coaching and medical follow-up care focused on lifestyle behavioural change in individuals with serious mental illness.	Mental Health	Prospective, longitudinal study	11 participants	Weight waist circumference blood pressure LDLs Triglycerides blood glucose levels Quality of life (Healthy Days Health-related Quality of Life questionnaire)	While some individuals showed improvement, others showed deterioration in the physiological markers for metabolic syndrome. Only a small number completed the 18-week study.	14
[38] Vietnam	To assess the feasibility of conducting a trial of a psychoeducational intervention involving the provision of tailored information and coaching to improve management of a cancer-related symptom cluster and reduce symptom cluster impacts on patient health outcomes in the Vietnamese context and to undertake a preliminary evaluation of the intervention.	Chronic illness	Parallel-group single-blind pilot quasi-experimental trial	102 cancer patients in one hospital	Numerical Analogue Scales for each symptom Brief Fatigue Inventory Pittsburgh Sleep Quality Index Karnofsky Performance Scale Hospital Anxiety and Depression Scale EuroQol-5D-5 L Intervention Rating Profile-15	The intervention group showed a significant reduction in symptom cluster severity, fatigue severity, fatigue interference, sleep disturbance, depression, and anxiety.	15
[5] (related to [9]) Finland	To evaluate a cost-effective analysis of a tele-based health coaching intervention among patients with type 2 diabetes, coronary artery disease, and congestive heart failure.	Chronic illness	RCT	998 participants with type 2 diabetes, coronary artery disease, or congestive heart failure	Health-Related quality of life Cost data: social and healthcare services	Cost effectiveness of the health coaching was highest in type 2 diabetes group. The probability of health coaching being cost effective was 55 % in the whole study group. Health coaching improved the quality of life for type 2 diabetes and coronary artery disease patients with moderate cost.	17
[1] Korea	To examine the effectiveness of a health coaching self-management program for older adults with multimorbidity in nursing homes	Chronic illness	RCT	43 older adults with multimorbidity in nursing homes	Self-management behaviours Self-efficacy Health status Chronic Disease Self-Management Program Questionnaire (42 items) Health goal setting and attainment scales (intervention group only)	Intervention group had better exercise behaviour, cognitive symptom management, mental stress management/relaxation, self-rated health, reduced illness intrusiveness, depression, and social/role activities limitations. Improved oral health and stress reduction.	15
[9] Finland	To evaluate the effect of a 12-month individualized health coaching intervention by telephone on clinical outcomes.	Chronic illness	An open-label cluster-randomized parallel groups trial	1221 participants with type 2 diabetes, coronary artery disease or congestive heart failure, and unmet treatment goals	Systolic and diastolic blood pressure serum total and LDL cholesterol concentration waist circumference for all patients, HbA1c	The diastolic blood pressure decreased to 85 mmHg or lower (48 % in the intervention group and 37 % in the control group). No significant differences emerged between two groups in the other primary outcomes. However, the target levels of systolic blood pressure and waist circumference were reached non-significantly more frequently in the intervention group.	16
[39] USA	To evaluate the effectiveness of transitional care coaching intervention offered to clinically ill medical patients during the transition from hospital to home (primary care).	Chronic illness	2 arm randomised pilot study; experimental post-test only	88 participants (60 intervention; 20 control)	Brief literacy measure Morisky Medical Adherence Scale Medication discrepancy tool	At home setting, many participants were unable or unwilling to discuss about goal setting and behaviour change. Those who were not able to participate had multiple distractions.	8
[40] UK	To test the effect of a telephone health coaching service (Birmingham Own Health) during primary nursing care on hospital use and associated costs.	Chronic illness	Retrospective design using person level administrative data and difference-in-difference analysis with matched controls.	2698 patients recruited from local general practices before 2009 with heart failure, coronary heart disease, diabetes, or chronic obstructive pulmonary disease	Hospital bed days Elective hospital admissions Outpatient attendances Secondary care costs	Emergency admission rates and outpatient attendance rate increased rapidly in intervention group.	17
[41] USA	To test the implementation of nurse-telephone coaching for families of children with asthma.	Chronic illness	RCT	12 families, 175 participants	4 targeted behaviours (Controller medications; asthma action plan; rescue meds; planning visits) Interview with parents	Nurse telephone coaching was successful in promoting improved asthma self-management behaviours in parents of children with asthma.	13
[42] USA	To test the effectiveness of two interventions compared to usual care in decreasing attitudinal barriers to cancer pain management, decreasing pain intensity, and improving functional status and quality of life.	Pain	RCT	318 adults with various type of cancer-related pain	Pain intensity Pain relief Pain interference Attitudinal barriers Functional status Quality of life	Attitudinal barrier scores did not change over time among groups. Patients randomised to the coaching group reported significant improvement in their ratings of pain-related interference with function, general health, vitality, and mental health.	15
[43] USA	To determine the efficacy of the Power Over Pain-Coaching intervention to improve functional status among African American outpatients with cancer pain.	Pain	Two-group randomised design with repeated measures	310 African American cancer patients	Pain Pain-related distress Functional status Perceived control over pain	Functional status improved. Distress also was differentially decreased. Pain intensity ratings decreased. The largest effects were observed in the living with pain component.	16
[44] Australia	To evaluate telephone coaching undertaken by practice nurses in a randomised controlled trial of self-management support for people with type 2 diabetes.	Chronic illness	Grounded theory	14 coaching session by 6 GP employed practice nurses	-	Patient-participants had complex multiple medical conditions to manage while maintaining daily lives. Two approaches to working with this complexity: treat to target; and personalised care.	14

*CASP* Critical Appraisal Skill Program, *GP* General Practice, *LDL* Low-density lipoprotein, *RCT* Randomised Controlled Trial, *UK* The United Kingdom, *USA* The United States of America

### Data abstraction

Of the 17 included studies, there were 14 quantitative articles and three qualitative studies. Summaries of included studies is summarized in Table 1. This table also summarized main features of each study such as its research design, study location, and primary outcomes (Table 1).

It is worthy of note the following; [32] and [33] reported two phase of Patient Self-Management for chronic obstructive pulmonary disease (COPD) (PSM-COPD) trial. [32] reported experiences of being coached while [33] explored effectiveness of the intervention. Therefore, they were considered different studies. Similarly, [31] and [44] reported on two studies under the umbrella of the research program, Patient Engagement And Coaching for Health (PEACH) project [44] explored participants’ views on managing their chronic symptoms whilst [31] evaluated the effectiveness of health coaching in a healthcare system. Therefore, these studies were included as two studies.

### Narrative synthesis

Each article was read multiple times to gain an in-depth understanding of the content in preparation for the process of abstracting key data relevant to the questions informing this review. The process by which this was undertaken was discussed by the authors prior to the data abstraction process. Two authors independently analysed the data. Any disagreements were discussed until consensus was gained.

A thematic analysis framework as guided by [28] was used to explore health coaching as provided by the Registered Nurses. There are six steps in this framework summarized in Table 2.

**Table 2:** Steps of thematic analysis as guided by [28]

1. Researcher familiarising themselves with the data
2. Generate initial codes
3. Search for themes
4. Review emerged themes – Generate thematic map
5. Defining and naming each themes and sub-themes
6. Select exemplar

## Results

### Evidence that nurses use coaching

Applying the chosen definition [6] was important during the analysis of this inquiry as this showed that Registered Nurses do use coaching. A list of the features from the definition [6] was identified and each included article was examined to see if, and which features of coaching by nurses were used in that particular research coaching intervention. A conclusion was made that Registered Nurses do use coaching in their practice which included primary care, aged care, and acute care settings (Table 3).

**Table 3:** Summary of health coaching interventions

References	Coaching strategy/intervention used	Preparation of coach/es	No. of coach/es	Coaching style	Interval of intervention	Length of intervention	Duration of each session
[31]	Goal-focused Treat to target (directive) Personalised care (non-directive)	Practice Nurses employed at GP practices	70	1 face-to-face 8 telephone	6 weekly (6 months) 2 months (6–12 months) 12 months 15 months	18 months	10–120 min per session Average 30 min
[32]	Based on Bandura’s Social Cognitive Theory Focused on smoking cessation services, increasing physical activity, medication management and action planning	Specially trained study nurses 2 days training	8	4 telephone	Week 1, 3,7,11	24 weeks	35–60 min (week 1) 15–20 min (week 3,7,11)
[34]	Motivational Interviewing	Registered nurses Certified in motivational interviewing	Not specified	6 telephone	biweekly	9 months	Not specified
[35]	DVD program (24 min) Management Motivational enhancement Identifying desired and attainable behavioural goals Behaviour plan	Trained diabetes nurse Bilingual nurse educator trained in patient-centred approaches	1	5 telephone	Case by case	6 months	15–60 min per session
[33]	Based on Bandura’s Social Cognitive Theory Focused on smoking cessation services, increasing physical activity, medication management and action planning	Specially trained study nurses 2 days training	8	4 telephone	Week 1, 3,7,11	11 weeks	35–60 min (week 1), average 39 min; 15–20 min (week 3,7,11)
[36]	Focus on giving information, interpreting the experience, and validating and clarifying responses and actions related to the surgical experience directed toward marking a difference in recovery outcomes	Minimum with Bachelor of Nursing degree 3 × 2-hour formal classes including the procedure for data collection for both the NCI and the UP groups, study instruments, data management, and, if interested, data entry.	12	4 telephone	1st night, 24, 48, and 72 h post-surgery	1 week	15–35 min per session
[37]	Motivational Interviewing Health promotion	Masters prepared Over 20 years of experience in psychiatric mental health nursing All attended motivational interviewing training	5	5 face-to-face	Week 2, 6, 10, 14, 18	18 weeks	-
[38]	Based on Revised Symptom Management Conceptual Model and the Individual and Family Self-Management Theory Self-management Knowledge and perceptions to motivate people to engage in desired symptom management behaviours, skills, and resources necessary to perform behaviours and support from family and health professionals to continue the behaviours. Individualized psychoeducational sessions	Experienced nurses	1	1 face-to-face 2 telephone	Weekly	3 months	1 h per session
[5]	Based on Self-Regulation Theory Techniques incorporated: Goal setting, motivational interviewing, action planning, active listening and open-end questioning	Experienced certified nurses and public health nurses Trained for 4 weeks in a telephone coaching model developed by Pfizer Health Solutions and modified for Finnish system. Two supervised sessions. Trained for motivational interviewing technique and telephone coaching	8	10–12 telephone	Monthly	12 months	30–60 min per call
[1]	3-level approach (individual, group, and facility) *Group level*: health education providing knowledge and motivating self-management behaviours *Individual level*: Goal setting	Training given by Principle investigator Geriatric nurse practitioners (2 experts in geriatric nursing and motivational interviewing) Nurses were divided into two groups (individual level or group level)	8	Group workshop Face-to-face	Weekly	8 weeks	1-hour group coaching 30 min per session
[9]	Based on Self-Regulation Theory Techniques incorporated: Goal setting, motivational interviewing, action planning, active listening and open-end questioning	Experienced certified nurses or public health nurses. Trained for 4 weeks in a tele-coaching model initially developed by Pfizer Health Solutions then modified for Finnish system. Two supervised sessions.	7	10–11 telephone	Monthly	12 months	30–60 min per call
[39]	Coaching framework based on appreciative inquiry theory (goal setting, achievement) 1 inpatient interview 1 48-hour post-discharge phone call 1 home visit/participant within 14 days of discharge 30, 60, and 90-day follow-up calls	Master’s prepared-RN Certified health coach	1	1 face-to-face 1 telephone 1 face-to-face 3 telephone	Immediately post-surgery 48-hour post-discharge 14, 30, 60, and 90 days post-discharge	3 months	
[40]	Personalised care plan Areas of coaching based on 8 priorities	Specially trained nurses employed by NHS Direct Content of training not specified	Not specified	12 telephone	Usually monthly (depending on patients’ situations)	12 months	15 min per call
[41]	Based on James Prochaska’s transtheoretical model of behaviour change Targeted 4 desired asthma care behaviours	Paediatric nurses working at St. Louis Children’s Hospital telephone triage service (registered nurse for at least 10 years, and an average of 5 years of paediatric telephone triage experience) Training was composed of two, 90-minute group sessions within a two-week period to review study design. Course content included an introduction to asthma coaching and the conceptual model, and review of documentation expectations. Coaches learned how to “stage” parents on their readiness to apply each of the targeted behaviours, then observed and practiced staging through role-playing and written assignments. They learned how to provide tailored care advice appropriate to each parent’s stage of readiness guided by a computerized protocol.	13	1–11 telephone	Average 4–8 calls	12 months	10 min per call
[42]	Motivational interviewing	Trained extensively in motivational interviewing and change theory by a cognitive behavioural psychologist and then in procedures related to the specific coaching protocol.	1	4 telephone	Fortnightly	6 weeks	30 min
[43]	Medication management Pain advocacy Living with pain	Master’s-prepared RN with prior experience in oncology or pain management and received additional training and monitoring by the principle investigator.	Not specified	3 face-to-face 2 telephone	2, 3, 4, 5 and 6th week	12 weeks	45–60 min (face-to-face); 10–15 min (telephone)
[44]	Lifestyle and pharmacological management: Five stages along a continuum of motivational readiness to engage in a healthy behaviour (Precontemplation; Contemplation; Preparation; Action; and Maintenance)	Licensed practice nurses who had extensive previous experience with asthma assessment and treatment Attended 5 training days Trained 2 days to deliver telephone coaching session to individuals Practice nurses employed by GP clinics	2	1 face-to-face 8 telephone	6 weekly (6 months) 2 monthlies (6–12 months) 12 months 15 months	18 months	-

*GP* General Practice, *NHS* National Health Service, *RN* Registered Nurse

All included articles in this review explored how coaching was implemented by Registered Nurses which answers the first question of this inquiry; “how do nurses coach?”

### Describing coaching interventions

#### Coaching interventions

Following section summarized the findings in relation to the types of coaching interventions used for health coaching (Table 3).

#### Number of coaches

The number of coaches used is important for quality assurance of the coaching intervention. The number of coaches involved in each study varied between studies. Of 17 articles included, there were three articles [34, 40, 43] which did not specify the number of coaches that were involved. Of those studies which stated number of coaches (14 studies), the range of number of coaches involved was from one coach [35, 38, 39, 42] to maximum of 70 coaches [31]. Most authors did not explain how they assessed the coaching education and training of Registered Nurses who provided the coaching and no study noted quality assurance procedures between coaches. Lack of quality assurance between coaches means a risk of inconsistent application of coaching which may have influenced study outcomes.

#### Length of intervention

The length of intervention varied from one week for post-surgery to 18 months for chronic illness management. The average duration of intervention across the studies was eight months. Number of coaching sessions offered varied from three to 12 sessions with average of seven coaching sessions provided throughout the duration of the coaching intervention. The length of time coaching is implemented is significant as time is required for those receiving coaching to identify desired goals, to determine strategies useful to reach these goals and to practice new lifestyle behaviours.

#### Duration of each coaching session

The duration of each coaching session varied between studies, with a minimum of ten-minutes to the maximum of 120-minutes. Of 17 studies, four studies [34, 37, 39, 44] did not report the duration of each session. In all the studies, which specified the duration of their coaching sessions, first coaching session are likely to be longer in comparison to follow-up or consecutive sessions. The duration of coaching sessions was also likely to be shorter (10–20 min) when they used telephone coaching. Duration of coaching is important to achieve the key areas involved in coaching. These include but are not limited to rapport building, identifying the need to be coached, setting the goals, and determining possible strategies to implement to ensure successfully achieving the proposed goal.

#### Preparation of coaches

Education and training of nurses prior to coaching was seen as a variable that may explain why some studies showed or did not show efficiency for coaching to improve the chronic conditions. Coaching is not a regulated practice at this point of time and therefore coaching generally has a range of education and training courses, with many not providing education through formal qualifications. Only one study [39] noted previous training before the study commenced. [39] reported that a nurse who provided the intervention (appreciative inquiry) was a master’s prepared Registered Nurse who was also a certified health coach. However, [39] did not define what was meant by ‘certified health coach’.

A number of studies included in this inquiry did provide a brief period of training in coaching. The length of specific coaching training for nurses prior to the intervention varied between studies. [32] trained nurses for their coaching intervention for two days using Social Cognitive Theory. In comparison, the longest training for nurses was provided by [5] who trained nurses for four-weeks in relation to telephone coaching. No information was provided about who provided the training. Some studies trained their coaches with additional skills such as motivational interviewing and emphasized the need for a patient-centred approach.

All coaches were Registered Nurses and therefore were well educated in the notion of person-centred care (sometimes referred to as patient-centred care, client-focused care or partnerships). This knowledge may have assisted the Registered Nurses to facilitate a patient-led approach to goal setting. Bachelor of Nursing degrees also provide the necessary communication and interpersonal skills training needed such as rapport building and active listening.

For many studies Registered Nurses were chosen to be coaches as they had specialized in a particular health area, such as diabetes, cardiovascular disease, mental illness, aged care, or oncology. For example, coaches in [35] study usually provided care for those with diabetes, [37] used coaches who had over 20 years experienced in psychiatric mental health nursing, [1] employed nurses trained for geriatric nursing but also provided training in motivational interviewing in their study, and [41] involved nurses who had trained in paediatric nursing. Having expertise in the area is similar to sports coaching where previous successful strategies are used multiple times to refine another person’s ability to perform.

Health Management area using nurse coaching.

The second question of this inquiry was “why do nurses coach?” Improving self-care was the reason why coaching was implemented by Registered Nurses with the most common reasons to do so being to prevent or manage a chronic illness (Table 4). Two main approaches to achieve enhanced health were used. Some studies focused on symptom management of a chronic condition. Patient reported symptoms that commonly impact on a person’s ability to function daily and subsequently affecting quality of life was a significant reason why coaching was used. Other studies focused on changing lifestyle behaviours (habits) such as diet and physical activity to either improve health or to prevent poor health. Some studies included both symptom management and improving lifestyle behaviours to enhance health.

**Table 4:** Why coaching was implemented

Study	Health Area	Specific Area targeted
[31]	Type II diabetes	Improving glycaemic control in patients
[34]	Type II diabetes	Improving lifestyle behaviours targeting nutrition and sleep.
[35]	Type II diabetes	Improving lifestyle behaviour changes improving nutrition and physical activity.
[33]	COPD	Improving lifestyle behaviours targeting increased physical activity, smoking cessation, and improved psychological health. Also enhancing self-efficacy in chronic illness management.
[36]	Post-operative care	Reducing anxiety in patient and family and increased functional status for the patient.
[37]	Preventative chronic illness in mental health patients	Improving lifestyle behaviours targeting nutrition for reductions in weight, Blood pressure, triglycerides and blood glucose through behaviour.
[38]	Cancer	More efficient self-care symptom management
[1]	Aged care	Improving self-management targeting increased exercise, cognitive activities and cooperation.
[9]	Chronic conditions	Improving lifestyle behaviours and management of the chronic illness and patient’s preferences. Also building on strengths and overcoming obstacles.
[42]	Cancer	Symptom management targeting pain, increasing functional status and improving quality of life.
[43]	Cancer	Symptom management targeting pain, in particularly medication management, perceived control of pain and living with pain.

The majority of studies (n=13) targeted chronic illness, either in chronic illness management or the prevention of developing a chronic illness. The greatest target group was those who had or were at risk of developing type II diabetes. The next most frequently presented area was pain management associated with cancer care. The last study used health coaching delivered by Registered Nurses for post-surgical care, specifically pain management.

For those studies who used health coaching by Registered Nurses to improve chronic conditions and/or avoid subsequent further comorbidities there was a range of outcome measures used to determine effectiveness of the intervention. Effectiveness of the coaching intervention was often measured by clinical markers. A frequently used clinical marker was blood sugar levels (BSL) for those with diabetes [31, 35] or those who were at risk of developing diabetes as a comorbidity [37]. Another clinical marker used was haemoglobin; specifically, if there was an increased level HbA_1c_ [9, 31, 35, 37]. Additional blood measures used included low-density lipoproteins (LDL)’s and triglycerides [9, 37].

Interestingly, the blood measures after nurse coaching had mixed results. Blood measures showed a significant improvement following coaching by [35], but not for [31] or [9]. Knight et al. [37] had mixed results with some in the intervention group showing improvement and others did not.

In addition to clinical markers, other health assessment strategies were used such as patient reported symptoms. This included self-reported symptoms by the patient like fatigue [38] and pain levels [42, 43]. Both studies using coaching for those in pain showed significant improvement in the participants’ ability to function whilst experiencing pain. Whilst [43] did find significant improvement in the level of pain, [42] concluded pain intensity scores at the end of their study were insignificant for the intervention group. Self-reported fatigue level was measured by [38] and after coaching this measurement had decreased. This may be explained by the reduction in sleep disturbances.

Five studies have discussed influence of coaching on mental health of individuals [1, 34, 36, 37, 42]. All studies conclude that participants who have received coaching had experienced reduced mental distress. For example, [36] conducted a randomised-control trial (RCT) of post-surgery patients and highlights individuals in intervention group (received coaching) reported significantly improved physical and mental health than those participants who have received usual care. Similarly, [1] implemented RCT of coaching in individuals living with comorbidities in a nursing home and concluded that participants in the intervention group had increased level of self-management and reduced mental stress level. Likewise, [42] also conducted RCT of participants who experienced cancer-related pain and suggested that individuals in the coaching intervention reported reduced level of pain and an increase in functioning, resulting in improved mental health. However, [37] had mixed outcomes where some participants, who are living with serious mental illness, had improved lifestyle behaviours while others deteriorated during the 18-weeks weekly coaching intervention.

It is argued by World Health Organization [45] and the Australian Institute of Health and Welfare [46] that changing to healthier lifestyle behaviours can improve health, including for those with chronic illness. Therefore, it is not surprising to find that Registered Nurses are using coaching with the goal to enhance lifestyle behaviours for their clients. The studies specifically relating to healthy lifestyle behaviour change focused on increasing physical activity and exercise (i.e. [33, 38]), self-care (i.e. [31, 37]), and dietary intake (i.e. [31, 36, 37]).

## Discussion

It is widely accepted that lifestyle behaviours are closely related to the prevention of chronic illness [47]. Lifestyle behaviours are habits that people develop over time such as types of food eaten, comfort eating during stress, sedentary or active lifestyles and stress management. Whilst this is well known, it has been challenging to consistently gain success by changing and sustaining the new healthier habit. Changing habits is complex and requires time. The challenge has been for researchers to define the time it takes to develop a complex habit, in this case changing to healthier lifestyle habits. The variable of time has been poorly defined across these studies with the lack of previous evidence for the justification for length of time used for the coaching intervention.

In addition to the ideal time to implement coaching, another important variable to consider is the number of habits to change. All studies included in this inquiry expected improvements in multiple lifestyle and self-care behaviours which may be a naive expectation. Lifestyle behaviours are habits that are typically associated with the issues of global ‘wicked problems’ like obesity and smoking [47]. The challenge of just changing one habit is often elusive and so to expect changes in multiple habits may have limited the efficacy of the coaching interventions of the included studies. This could explain why some coaching interventions did not appear to be effective, i.e. the intervention merely was not long enough for the old habit to be replaced by the new and developing habit or there were too many habits to work on simultaneously. Unfortunately, there is a death of evidence to guide coaching interventions about the length of time or the number of goals to set.

Outcome variables that show improvement from a coaching intervention is another area worthy of discussion. Biomarkers have dominated the measurement of the impact of coaching in contemporary studies. Overall, biomarkers have shown an improvement in a person’s condition in the short to medium term following coaching (although sometimes only slightly), which is promising. Yet more research to determine other suitable biomarker levels is needed. Other measurements that show new healthy habits are developing would also be useful such as from exercise science and occupational changes in daily activities.

As yet there is slight evidence to continue to pursue using coaching by Registered Nurses. Two recent studies have shown benefits for both then nurse coach and the client in self-development and self-reported improved self-care [48, 49]. What would aid this discussion is the cost of coaching. Questions can be asked about the feasibility of coaching according to cost. At the coaching site some may say the cost is not worth the small improvement in the health of the participants. However, additional ways to consider costs need to be examined. Worthy to note is that poor lifestyle behaviours leading to obesity and other conditions contributing to chronic illness is a great cost to society [50, 51]. Cost of benefits of a health coaching intervention addressing these “wicked problems” should be compared to the hypothetical economic costs if coaching is not used and these poor health conditions were sustained. This would include but is not limited to reduced health care costs, decreased sick leave, and subsequent increased productivity of society. If economic feasibility studies did show that coaching costs are minimal in comparison to sustaining the “wicked problems” then budgetary costs for a longer-term coaching intervention would be a worthy consideration here; meaning sociological cost may be a more meaningful measure to extend the coaching time.

It is not surprising that nurse coaches are being engaged for chronic illness management. Nurses have many professional skills like therapeutic communication including active listening, being opened to other people’s experiences and knowledge and skills associated with managing chronic illness. Coaching can include motivational interviewing, as well as goal setting and assessment of progress; all skills nurses can implement into their practice. However, nurse coaching is different to the usual practice of nursing. First coaching uses these skills in a systematic way with a specific purpose that may differ to the usual purposes of nursing care. Second, nurse coaching is not widely used.

The other strategy for working with patients noted in many of the studies was patient education. Nurse-led education and assessing for patient health literacy have become essential nursing strategies. Health literacy is a relatively recent area discussed in the literature and “… *entails people’s knowledge, motivation and competences to access, understand, appraise, and apply health information in order to make judgments and take decisions in everyday life concerning healthcare, disease prevention and health promotion to maintain or improve quality of life during the life course*” [52]. For studies wishing to examine efficiency and efficacy of nurse coaching to improve health one needs to differentiate from health education and literacy, and coaching. Some of the included studies in this inquiry did not differentiate between educating the patient versus coaching to improve self-care. Education should occur first and health literacy of the patients should be assessed prior to coaching commencing. Of course, patients could desire improved health literacy and set this as one of the goals to achieve during coaching but generally coaching time should not be taken up with educating the person.

The training provided to Registered Nurses before coaching was implemented varied across the studies included in this inquiry. This did create curiosity of what education was available to prepare Registered Nurses to coach. Formal education to Registered Nurses and other health professionals about coaching itself has only just commenced in Australian universities.

Gaining a degree in coaching at universities is relatively new. The most common degrees provided in the United States of America related to coaching tend to be exercise or sports coaching and less frequent, health coaching. The universities in the United Kingdom predominantly provide sports coaching with less providing degrees in business coaching. Universities in Asia focus on sports coaching. Universities in Europe predominantly also provide coaching for sports, with others offer some business and personal development coaching. Australia has commenced degrees in business coaching with some short courses in other types of coaching. However, coaching degrees based on high quality evidence is not commonly available across the globe and there is no obligation at this point to gain formal education as coaching is not regulated.

In the past coaching education has typically been provided by others who are untrained in education (other coaches who do not have professional qualifications). Many independent and commercial organizations do provide training in coaching and are not obliged to audit for effectiveness of their education or the competency of their graduates. They may use marketing to suggest coaching is transformative but lack research evidence of those claims. Even those that provide some structure to reviewing competency may do so without appropriate education and competency knowledge or research skills that would be useful to show if the training has successfully transitioned from education to practice. Whilst those involved with independent training organisations have positive intentions, the process they use for evaluation of their programs is merely superficial and not rigorous, and is often based on student satisfaction. Typically, these organizations and their training programmes are not audited independently by external others. This is a significance difference compared to universities who are obliged to conduct external audits. Such quality and assurance are important for all education, including coaching. An additional great concern is the lack of standards and regulations in the area of coaching.

Organizations have evolved to address the informality and lack of regulation in the area of coaching, and provide accreditation and membership for a professional body of coaches. Such organizations include but not limited to: the International Coaching Federation (ICF), the Association for Coaching (AC), and the European Mentoring and Coaching Council (EMCC) [53] argued for best practice and educational benchmarks. To reflect such standards they proposed a national certification for the role of a health and wellness coach. More recently, a specific nurse coach organization has been formed in the America, The International Nurse Coach Association and a specific nurse coaching program, the Integrative Nurse Coach Certificate Program has been piloted [48]. These organizations are noted as they aim for best practice and continuous improvement in coaching providing standards for coaching practice. In the quest to achieve professional standards, like many other contemporary disciplines who evolved over time, it will be important to recognize those before them who have paved the way in coaching. Considerations recognizing prior learning and experience when awarding individual accreditation is paramount at this time of coaching disciplinary development.

Extending the scope of nursing practice to routinely coach in self-care would be ideal, rather than such coaching be provided by unregistered others who have not been through an independent and systematic approach to being audited as conducting appropriate and safe coaching. It is important that coaching interventions are delivered in a safe and fruitful way for those who are often most vulnerable. Principles of beneficence and maleficence are fundamental to nursing practice, therefore equipping nurses to lead the way in health coaching.

### Limitations

This review was limited by exploring the topic of “health coaching” provided by Registered Nurses. Whilst health coaching was the obvious type of coaching nurses are likely to implement, it is acknowledged that Registered Nurses may use variety of coaching other than health coaching.

An additional limitation worth noting is the inability to differentiate that nurses used coaching as well as mentoring, or provided advisory information to the clients receiving the coaching intervention. Whilst we could show that the Registered Nurses implemented coaching, we could not confirm that they did not use mentoring and/or advisory information at the same time. There was some evidence they did provide education during the intervention (advisory information) but there was no evidence that they did also mentor. However, as these three approaches do differ, coaching, mentoring and the provision of advisory information, it is not possible to conclude that the coaching was undertaken alone and the other nursing strategies of mentoring and the provision of advisory information may have influenced the effectiveness (or lack of) coaching in these included studies.

## Conclusion: recommendation for practice and research

In conclusion, nurses should lead the way in the practice of health coaching. They are well placed to deliver coaching as coaching theories are typically aligned with theories nurses have been using for many decades. Clients should be cautioned about possible unsafe practice from untrained others using coaching without regulations. The registration of nurses means legal and regulatory obligations for safe practice with consequences if one does not practice appropriately. In comparison to others implementing health coaching who are not regulated, there is a lack of clarity of how a client could report unsafe or inhumane coaching. People with long term health issues are often most vulnerable and nurses are accustomed to providing ethical care to this group.

It remains a concern that there is no obligation at this point to gain formal education as coaching is not regulated. However, this means that Registered Nurses are ideally positioned to provide health coaching as they have foundational education in theories and practice that are necessary in health coaching. What would be useful is for targeted coaching education for Registered Nurses and other health professionals to optimise coaching strategies. This could include but is not limited to units of coaching offered in degrees and more postgraduate professional development courses. Regardless of what type of education Registered Nurses receive, it is recommended that coaching training should be based on research evidence, not just customer satisfaction for coaching as many unregulated education businesses tend to use.

Evidence for best practice when including nurse coaching is not yet conclusive. Clarity is required for evidence of the ideal frequency of coaching, number of coaching sessions required to make change, and evidence for “Best Training” for learning to coach. Further research to determine how coaching changes and sustains lifestyle behaviours would be ideal. Some studies did show improvement. Therefore, determining how long on average it does take coaching to change lifestyle behaviours, and how many behaviours can be changed at one time would be useful to know so that budgets were realistically determined. Additionally, more research which supports or refutes coaching in preventing ill health would be useful to determine the feasibility for nurse coaches to extend their coaching practice to increased prevention as well as managing chronic illness routinely.

Scope of nursing practice could also be reviewed. Registered Nurses should advocate for more coaching in areas like recovery from surgery and extend this to recovery from injuries. Registered Nurses working in general practice implementing coaching would be ideal to complement therapy from other health disciplines like physiotherapy and occupational therapy.

Finally, health education should be conducted prior to implementing health coaching to continue to facilitate health promotion for patients. This means the coaching time is not taken up with lessons on how to be healthy. Rather the time is used to motivate people through setting goals, strategizing ways to achieve these goals, to identify obstacles in implementing these strategies and to review progress to date. In this way, Registered Nurses can optimize the focus on motivating change or facilitating optimal self-care practice of patients. Nurse coaching adds another tool in the nursing competency toolbox. Once there is sufficient evidence-based knowledge coaching will be ideal to optimize patient health and wellbeing.

## Data Availability

All data generated or analysed during this study are included in this published article.

## Abbreviations

AC: Association for Coaching.
BSL: Blood Sugar Level.
CASP: Critical Appraisal Skilled Program.
CINAHL: Cumulative Index to Nursing and Allied Health Literature.
COPD: Chronic Obstructive Pulmonary Disease.
EMCC: European Mentoring & Coaching Council.
ICF: International Coaching Federation.
LDL: Low-density lipoproteins.
MeSH: Medical Subject Heading.
PEACH: Patient Engagement And Coaching for Health.
PRISMA: Preferred Reporting Items for Systematic Reviews and Meta-Analysis.
PSM-COPD: Patient Self-Management Chronic Obstructive Pulmonary Disease.
